# Anti-Oxidative and Cholinesterase Inhibitory Effects of Leaf Extracts and Their Isolated Compounds from Two Closely Related *Croton* Species

**DOI:** 10.3390/molecules18021916

**Published:** 2013-02-01

**Authors:** Ashwell R. Ndhlala, Mutalib A. Aderogba, Bhekumthetho Ncube, Johannes Van Staden

**Affiliations:** 1 Research Centre for Plant Growth and Development, School of Life Sciences, University of KwaZulu-Natal Pietermaritzburg, Private Bag X01, Scottsville 3209, South Africa; E-Mails: ndhlala@ukzn.ac.za (A.R.N.); Aderogba@ukzn.ac.za (M.A.A.); 209522727@stu.ukzn.ac.za (B.N.); 2 Department of Chemistry, Obafemi Awolowo University, Ile-Ife 220005, Nigeria

**Keywords:** acetylcholinesterase, antioxidant activity, β-carotene-linoleic, *Croton gratissimus*, *Croton zambesicus*, DPPH, Euphorbiaceae, FRAP, phospholipid peroxidation

## Abstract

A comparative evaluation of the antioxidant and acetylcholinesterase inhibitory activity of the leaf extracts of *Croton gratissimus* and *Croton zambesicus* (*subgratissimus*) and compounds isolated from the extracts was carried out to determine their potential and suitability or otherwise as a substitute for each other in the management of oxidative and neurodegenerative conditions. Different antioxidant assays (DPPH, FRAP, β-carotene-linoleic and the lipid peroxidation models) and the microplate assay for acetylcholinesterase (AChE) inhibition were carried out separately to study the activities of the crude leaf extracts and four solvent fractions from each of the two *Croton* species. Bioassay guided fractionation was used to target antioxidant constituents of the crude extracts and ethyl acetate fractions of 20% aqueous methanol extract of *C. gratissimus* on silica gel and Sephadex LH-20 columns resulted in the isolation of kaempferol-3-*O*-β-6’’(p-coumaroyl) glucopyranoside (tiliroside, **2**), apigenin-6-*C*-glucoside (isovitexin, **3**) and kampferol (**4**). The extract of *C. zambesicus* yielded quercetin-3-*O*-β-6’’(p-coumaroyl) glucopyranoside-3’-methyl ether (helichrysoside-3’-methyl ether, **1**), kaempferol-3-*O*-β-6’’(p-coumaroyl) glucopyranoside (tiliroside, **2**) and apigenin-6-*C*-glucoside (isovitexin, **3**). Three of the isolated compounds and their different combinations were also included in the bioassays. In all the assays performed, the antioxidant capacity and AChE inhibitory effects of *C. zambesicus* extracts were weaker than those of *C. gratissimus*. This suggests that *C. gratissimus* may not be substituted by *C. zambesicu*s, despite the similarity in some of their constituents. Generally, the combinations made from the isolated compounds showed better activities in most of the assays compared to the individual isolated compounds. This suggests mechanisms such as synergism and/or additive effects to be taking place. This study established low, moderate and high antioxidant activities as well as AChE inhibitory effects by the crude extracts, fractions, compounds and compound combinations. This means some of the extracts, isolated compounds and compound combinations could be useful in the management of neurodegenerative conditions and serve as sources of natural neurodegenerative agents.

## 1. Introduction

Oxidation reactions form an essential part of normal metabolism within living systems as oxygen is the ultimate electron acceptor in the electron flow system that produces adenosine-5’-triphosphate (ATP) [1]. Problems may arise when there is an overproduction of reactive oxygen species (ROS) due to the uncoupled electron flow and energy production, priming chain reactions which produce excess free radicals [2]. Excess free radicals within the body (oxidative stress) results in severe damage to biological molecules, especially to deoxyribonucleic acid (DNA), proteins and lipids [3]. Oxidative damages was used as the basis for the “free-radical hypothesis of aging” [3,4] and the “oxidation-stress hypothesis of atherosclerosis” [4,5]. Therefore, oxidative stress has been associated with the pathogenesis of many other diseases including diabetes mellitus, atherosclerosis, neurodegenerative diseases such as Alzheimer’s Disease (AD), different malignant diseases and virus infections, including HIV/AIDS [4,6].

The use of herbs in traditional medicine is a cost-effective system that offers therapeutic benefits that differs in substance, methodology and philosophy to modern medicine, and plays an important role in health maintenance for the peoples of the world, including those suffering from oxidative and neurodegenerative diseases [7]. Extensive use of herbs and their therapeutic effects has lead many research groups to examine the chemical nature, activity and mechanisms of action of natural healing compounds [8]. As a result, numerous secondary metabolites from medicinal plants have been reported to possess therapeutic properties. Among them, phenolic compounds, have been extensively examined as retardants in disease progression. This has generated a great number of articles, covering past and current knowledge mostly on the activity of food/herbal phenolic as natural antioxidants [9].

There is now increasing experimental and clinical evidence which shows that drug combinations might be a more effective strategy for therapy, given the multiplicity and complexity of most diseases. Drug interactions result in synergistic, indifferent, additive or antagonistic effects [10]. An advantage occurs when the activity of the combined compounds is greater than the individual contribution of each agent. The benefits of such synergy include a wider spectrum of efficacy, tolerability and a reduction in resistance (in the case of antibiotics) [10]. Some researchers have recommended intense studies involving plant combinations [11].

*Croton gratissimus* Burch (leaves with reddish scales below) and *C. zambesicus* Müll. Arg. *subgratissimus* belong to the family Euphorbiaceae. *C. gratissimus* is used to treat coughs, fever, abdominal disorders, respiratory disorders, skin inflammation, earache, malarial and chest complaints [11,12]. The leaf decoction of *C. zambesicus* is used in the management of type 2 diabetes mellitus, hypertension and malaria in Nigerian folk medicine [13,14]. The root and stem bark extracts demonstrated antiplasmodial activity [15,16].

Previous phytochemical investigation of the stem, bark and leaf extracts of *C. gratissimus* yielded cembranolides, some of which exhibited moderate activity against the progressive external ophthalmoplegia 1 (PEO1) and PEO1TaxR ovarian cancer cell lines as well as *in vitro* antiplasmodial screening against *P. falciparum* (CQS) D10 strain [12,17]. Leaf, stem and bark extracts of *C. zambesicus* afforded flavone-*C*-glycosides and diterpenoids [18,19,20,21,22].

This study was aimed at a comparative investigation of the anti-oxidative capacity and acetylcholinesterase (AChE) inhibitory properties of leaf crude extracts, solvent fractions, isolated compounds and different combinations of the isolated compounds from *C. gratissimus* and *C. zambesicus* using different antioxidant assays and the microplate technique for AChE inhibition. This will also provide information on whether the two closely related species can be interchangeably used in folk medicine.

## 2. Results and Discussion

**Figure 1:**
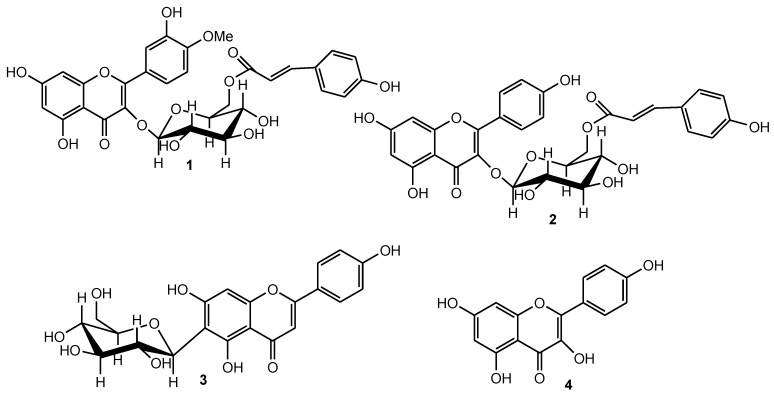
Compounds isolated from *Croton gratissimus* and *Croton zambesicus.* Quercetin-3-*O*-β-6’’(p-coumaroyl) glucopyranoside-3’-methyl ether (helichrysoside-3’-methyl ether, **1**), kaempferol-3-*O*-β-6’’(*p*-coumaroyl) glucopyranoside (tiliroside, **2**), apigenin-6-*C*-glucoside (isovitexin, **3**) and kampferol (**4**).

In our efforts to find phytochemical agents that could be effective in the prevention and management of neurodegenerative conditions, a comparative study was carried out on two closely related *Croton* species to determine their antioxidant and acetylcholinesterase potentials and suitability or otherwise as a substitute for each other in the management of neurodegenerative conditions. Bioassay guided fractionation was used to target antioxidant constituents of the crude extracts and ethyl acetate fraction of a 20% aqueous methanol extract of *C. gratissimus* on silica gel and Sephadex LH-20 columns afforded kaempferol-3-*O*-β-6’’(p-coumaroyl) glucopyranoside (tiliroside, **2**), apigenin-6-*C*-glucoside (isovitexin, **3**) and kampferol (**4**). The extract of *C. zambesicus* yielded quercetin-3-*O*-β-6’’(*p*-coumaroyl) glucopyranoside-3’-methyl ether (helichrysoside-3’-methyl ether, **1**), kaempferol-3-*O*-β-6’’(p-coumaroyl) glucopyranoside (tiliroside, **2**) and apigenin-6-*C*-glucoside (isovitexin, **3**). The structures of the isolated compounds were elucidated using spectroscopic techniques and are presented in Figure 1.

### 2.1. DPPH Radical Scavenging Assay

The concentration of a test sample needed to decrease the initial DPPH concentration by 50% (EC_50_) is a parameter widely used to measure antioxidant activity [8]. The EC_50_ values for the DPPH radical scavenging potentials of the three isolated compounds, four different compound combinations, crude extracts and four solvent fractions from *C. gratissimus* and *C. zambesicus* are shown in Table 1. In this study, EC_50_ values less than or equal to 70.12 µg/mL (obtained for the positive control) were considered good activity. The order of potency in the radical scavenging activity against DPPH radicals according to the respective EC_50_ values (Table 1) that were less than 70.12 µg/mL were as follows: *C. gratissimus* butanol fraction > *C. gratissimus* ethyl acetate > *C. gratissimus* crude extract > combination 3 (helichrysoside-3’-methyl ether + isovitexin) > combination 2 (helichrysoside-3’-methyl ether + tiliroside).

Generally, the compound combinations showed better scavenging activity when compared to each of the isolated compounds. This suggests mechanisms such as synergism and/or additive effects as being responsible. On the other hand, *C. zambesicus* extracts were less active in the scavenging power compared to *C. gratissimus*. There are several mechanisms by which antioxidants exert their scavenging properties. In all the mechanisms, scavenging of radicals depends on the rate of hydrogen atom transfer from the compounds to the radicals [23].

### 2.2. β-Carotene-Linoleic Acid Model System (CLAMS)

The results for the prevention of oxidation of *β*-carotene and linoleic acid in a model system by the test compounds and extracts are presented in Table 1. The presence of an active antioxidant delays the rate of *β*-carotene bleaching. Heat (50 °C)-induced oxidation involves the subtraction of a H-atom from an active methylene group of linoleic acid, forming a linoleate free radical. The linoleate radical then viciously attack the highly unsaturated *β*-carotene in an effort to regain its lost H-atoms. As the *β*-carotene is attacked, it loses its orange colour. The presence of a good antioxidant can prevent the attack on *β*-carotene by neutralizing the linoleate radical. In this study, the delay in *β*-carotene bleaching was recorded as antioxidant activity (ANT %), calculated on the basis of the rate of *β*-carotene bleaching at time = 90 min. Variable antioxidant percentages (ANT), shown in Table 1, were observed for the test compounds and extracts with *C. gratissimus* ethyl acetate fraction showing the highest percentage activity (96.66 ± 2.067%) and *C. gratissimus* DCM fraction the least (67.12 ± 0.99%).

Another antioxidant factor, Oxidation Rate Ratio (ORR) at time = 90 min was also determined (Table 1). Lower ORR values, just like EC_50_ values, denote better antioxidant potentials. Based on ORR, the order of antioxidant capacity with respect to the protection of *β*-carotene against bleaching by the compounds and extracts less than or equal to that of ascorbic acid (0.19) was as follows; *C. gratissimus* ethyl acetate fraction > *C. gratissimus* butanol > *C. gratissimus* crude extract (20% MeOH) = tiliroside > *C. zambesicus* ethyl acetate > *C. zambesicus* butanol > combination 2 (helichrysoside-3’- methyl ether + isovitexin) > combination 1 (helichrysoside-3’-methyl ether + tiliroside + isovitexin) > *C. zambesicus* crude extract (20% MeOH) = helichrysoside-3’-methyl ether > *C. gratissimus* hexane.

The ability to delay bleaching of *β*-carotene depends on the nature of the phenolic compounds. Flavonoids have greater ability to delay bleaching of *β*-carotene than other phenolic compounds [24]. Flavonoids reduce free radicals by quenching, up-regulating or protecting antioxidant defences and chelating radical intermediate compounds. This group of compounds has also been shown to inhibit the enzymes responsible for free radical production, for instance xanthine oxidase, cyclooxygenase, lipoxygenase, microsomal monooxygenase, glutathione *S*-transferase, mitochondrial succinoxidase, NADH oxidase and protein kinase C [25]. Of the three compounds tested in this study, tiliroside (**2**, Figure 1), isolated from both *C. gratissimus* and *C. zambesicus*, exhibited the best activity.

### 2.3. Ferric-Reducing Power Assay

**Figure 2:**
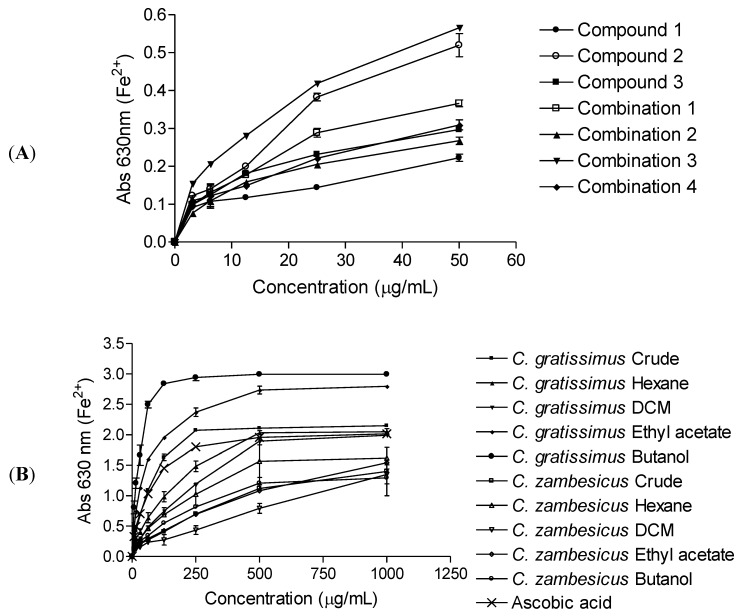
Ferric reducing activity of the compounds, crude extracts and fractions from *C. gratissimus* and *C. zambesicus*. (**A**); Helichrysoside-3’-methyl ether (**1**), tiliroside (**2**), isovitexin (**3**), combination 1 (helichrysoside-3’-methyl ether + tiliroside + isovitexin), combination 2 (helichrysoside-3’-methyl ether + tiliroside), combination 3 (helichrysoside-3’-methyl ether + isovitexin), combination 4 (tiliroside + isovitexin). (**B**); Crude extracts and fractions. Increase in absorbance of the reaction mixture indicates the increase in reducing power. Values represent mean ± standard error (n = 3).

Figure 2 presents the abilities of the isolated compounds (**A**), crude extracts and fractions (**B**) at varying concentrations to reduce Fe^3+^ solution. There were notable increases in absorbance as the concentrations of the test samples were increased. Depending on the reducing power of the test sample, the initial yellow colour of the reaction mixture changes to various shades of green and blue. A strong antioxidant (reductant) reduces the Fe^3+^/ferricyanide complex to a green/blue ferrous form, exhibited by higher absorbance values at λ 630 nm.

Combination 3 (helichrysoside-3’-methyl ether + isovitexin) was the best reducing agent, followed by tiliroside (**2**). For the crude extracts and fractions, the *C. gratissimus* butanol fraction showed the best reducing powers. As observed in the DPPH and CLAMS assays discussed above, the activity of *C. gratissimus* extracts as reducing agents were better compared to those of *C. zambesicus.* The reducing powers of the test samples were probably due to the electron donating abilities of the compounds and extracts [23]. The compounds could react with free radicals, by donating electrons, thereby converting them to more stable products and terminate radical chain reactions.

The reducing powers of the extracts were probably due to the electron donating abilities of the compounds [23]. The isolated compounds could react with free radicals to convert them to more stable products and terminate radical chain reactions.

### 2.4. The Phospholipid Peroxidation Assay Coupled with the Thiobarbituric Acid-Malondialdehyde (TBA-MDA) Model System for Oxidative Protection

**Figure 3:**
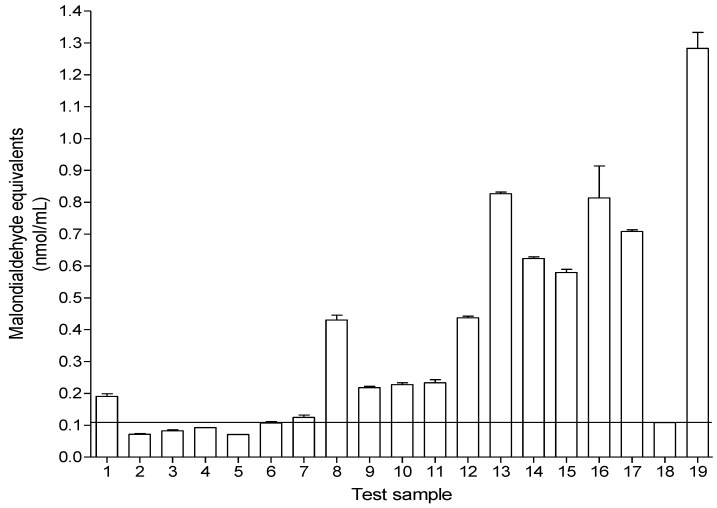
Malondialdehyde (MDA) equivalents (nmol/mL) recovered from the peroxidation of phospholipid in the presence of test samples. Isolated compounds were tested at 350 µg/mL while the crude extracts, fractions and ascorbic acid were tested at 3.5 mg/mL. Helichrysoside-3’-methyl ether (**1**), tiliroside (**2**), isovitexin (**3**), combination 1 (helichrysoside-3’-methyl ether + tiliroside + isovitexin) (**4**), combination 2 (helichrysoside-3’-methyl ether + tiliroside) (**5**), combination 3 (helichrysoside-3’-methyl ether + isovitexin) (**6**), combination 4 (tiliroside + isovitexin) (**7**), *C. gratissimus* crude 20% methanol extract (**8**), *C. gratissimus* hexane fraction (**9**), *C. gratissimus* DCM fraction (**10**), *C. gratissimus* ethyl acetate fraction (**11**), *C. gratissimus* butanol fraction (**12**), *C. zambesicus* crude 20% methanol extract (**13**), *C. zambesicus* hexane fraction (**14**), *C. zambesicus* DCM fraction (**15**), *C. zambesicus* ethyl acetate fraction (**16**), *C. zambesicus* butanol fraction (**17**), ascorbic acid (positive control) (**18**) and water (negative control) (**19**). Lower amounts of MDA, signifies higher protective ability of extract against lipid peroxidation. Values represent mean ± standard error (n = 2).

The ability of the test samples to inhibit and/or protect against the peroxidation of phospholipids is shown in Figure 3. In biological systems, lipid peroxidation generates a number of degradation products such as malonyldialdehyde (MDA). MDA is found to be the most important cause of cell membrane destruction and cell damage [26]. The formation of MDA, a secondary product of oxidative lipid degeneration, has been used as an index of lipid peroxidation and oxidative stress [27]. In this study, the ability of the test extracts to prevent lipid peroxidation was followed using a system that contained rat brain homogenates whereas peroxidation was induced by addition of a Fe complex. At the end of the reaction, the amount of MDA recovered signified the degree of lipid peroxidation. Lower amounts of MDA signifies higher protective ability of extract against lipid peroxidation.

The protective effects against oxidative stress by preventing lipid peroxidation of compounds **2** and **3** (tiliroside and isovitexin respectively) as well as combinations 1, 2 and 3 [combination 1 (helichrysoside-3’-methyl ether + tiliroside + isovitexin), combination 2 (helichrysoside-3’-methyl ether + tiliroside) and combination 3 (helichrysoside-3’-methyl ether + isovitexin)] were more than that of ascorbic acid, as signified by the lower amounts of MDA recovered. The crude extracts and the fractions exhibited weak protective effects against lipid peroxidation. Combining helichrysoside-3’-methyl ether and tiliroside resulted in the best protective effect against lipid peroxidation (Figure 3).

Enhanced peroxidation and oxidative stress can damage membrane lipids, DNA and functional proteins. The oxidation and peroxidation within the living systems are caused by excess reactive oxygen species (ROS) [23]. Over production of ROS such as superoxide anion radical, hydrogen peroxide and hydroxyl radical production occurs primarily as by-products of cellular metabolism in the mitochondria and normal mitochondrial respiration [28]. The compounds, some extracts and fractions reported here have demonstrated the ability to protect against such damage, especially when used in combinations.

### 2.5. Acetylcholinesterase (AChE) Enzyme Inhibitory Bioassay

The results of AChE inhibitory activity are presented in Table 2. The activity of three of the isolated compounds, the four compound combinations and the fractions varied from low to moderate except for *C. gratissimus* ethyl acetate and butanol fractions which had IC_50_ values less than 100 µg/mL (66.9 and 64.5 µg/mL, respectively). Isovitexin exhibited a better AChE activity compared to the other two compounds (**1** and **2**). This could be because of the position of the sugar moiety in isovitexin which leave the hydroxyl and ketone groups readily available for some reactions. The position of the sugar moiety in tiliroside (**2**) and helichrysoside-3’-methyl ether (**1**) presents some steric hindrance, partially preventing the compounds to either fit into the active site or making the hydroxyl and ketone groups not readily available for reactions.

The crude extract and fractions of *C. gratissimus* were more active than those of *C. zambesicus*. Generally, as in the other assays, compound combinations presented better inhibitory percentages and IC_50_ values compared to the individual compounds. The best combination (3) was that of tiliroside and isovitexin which exhibited an IC_50_ value of 141.7 µg/mL. The search for new inhibitors of AChE derived from natural sources, with fewer side effects is urgently required [29]. The progression and cognitive impairment of Alzheimer’s disease (AD) has been associated with oxidative stress in elderly persons as well as depression and anxiety amongst the general population. It is therefore, a bonus to get natural compounds that act both as antioxidants and AChE inhibitors, such as the isolated compounds and fractions reported from these two *Croton* species.

It has been observed that combining the isolated compounds studied here often resulted in activity close to one of the compounds or better than the individual compounds. As mentioned before, antioxidants exert their function by the rate of hydrogen atom transfer from the compounds to the radicals. The isolated compounds investigated here are phenolic in nature and the antioxidant properties of plant extracts have been attributed to their phenolic constituents. It has been reported that the multiple hydroxyl groups in the chemical structure of phenolic compounds make them ideal for free radical-scavenging reactions and the arrangement of the hydroxyl groups around the phenolic molecule is also important for antioxidant reactions. Relating the structure and function of the compounds studied here, *i.e.*, mixing helichrysoside-3’-methyl ether (**1**), and isovitexin (**3**) (Figure 1) in different combinations resulted in complexes with more hydroxyl groups, strategically arranged for antioxidant reactions. As reported by Hagerman *et al*. [30], the antioxidant potential of phenolic compounds improves as the number of hydroxyl groups increases, hence the higher antioxidant ability of condensed and hydrolyzable tannins at quenching peroxyl radicals over simple phenols. In all the assays, tiliroside (**2**) has been found to be a more active compared to helichrysoside-3’-methyl ether (**1**), regardless of their closely related structures. It is interesting to note how substitution of a methyl group with a hydroxyl group in helichrysoside-3’-methyl ether at carbon number 4’ to obtain tiliroside can significantly alter the activity of a bioactive compound. Research into the use of combined drugs is gaining more attention as it is apparently known, through observation, that many diseases possess a multi-causal etiology and a complex pathophysiology. The use of combinational drugs can be used more effectively in managing oxidative conditions compared to single drug therapies. Combinational drug therapy is currently practiced worldwide in the treatment of tuberculosis, HIV/AIDS symptoms and other infectious diseases, hypertension, cancer and rheumatic diseases.

In all the assays performed in this study, the antioxidant capacity and AChE inhibitory effects of *C. zambesicus* were weaker than that of *C. gratissimus*. This suggests that *C. gratissimus* may not be substituted by *C. zambesicu*s, despite the similarity in some of their constituents. Some compounds present in *C. gratissimus* could be playing a part in the activity demonstrated by the crude extracts and fractions in the form of synergism or additive effects. The concentration of the active compounds could also be different in the extracted materials. This can be due to different environmental conditions under which the plants grew. *C. zambesicu*s was obtained from Nigeria, while *C. gratissimus* was collected from South Africa. Different climatic conditions and/or soil fertility status can influence plant secondary metabolite dynamics.

## 3. Experimental

### 3.1. General

Acetylthiocholine iodide (ATCI), 2,2-diphenyl-1-picrylhydrazyl (DPPH), galanthamine, 5,5-dithiobis-2-nitrobenzoic acid (DTNB), AChE enzyme (isolated from electric eels) (type VI-S lyophilized powder) and β-carotene were obtained from Sigma-Aldrich (Sigma Chemical Co., Steinheim, Germany); Potassium ferricyanide from BDH Chemicals Ltd (Poole, England); trichloroacetic acid, ascorbic acid, polyoxyethylene sorbitan monolaurate (Tween 20), ferric chloride (FeCl_3_) and methanol from Merck KGaA (Darmstadt, Germany). All other chemicals used in the assays were of analytical grade. All thin layer chromatography analyses were performed at room temperature using pre-coated plates (Merck, silica gel 60 F_254_ 0.2 thickness). Detection of spots was done by viewing under ultraviolet light (254 and 365 nm). Open column chromatography was carried out using Sephadex LH-20. Nuclear magnetic resonance (NMR) data were obtained on a Bruker 400 MHz spectrometer. Chemical shifts are expressed in parts per million (ppm).

### 3.2. Plant Collection and Authentication

The leaves of *C. zambesicus* were collected at the Department of Cultural Studies, Obafemi Awolowo University, Ile-Ife, Nigeria, in August, 2012. The plant was identified by Mr. O. Oladele of the Herbarium Section, Faculty of Pharmacy, Obafemi Awolowo University. The leaves of *C. gratissimus* were collected at a private residence, Alan Paton Drive, Pietermaritzburg. It was identified by Mrs A. Young (Horticulturist), of the Botanical Garden, Life Sciences Department, University of KwaZulu-Natal, Pietermaritzburg, South Africa. Herbarium specimen (Aderogba MA 02) was deposited at the Bews Herbarium (NU). The collected plants were separately oven dried at 40 °C for 3 days and ground to powders.

#### 3.2.1. Extraction

The powdered plant material (1 kg) of *C. gratissimus* was extracted with 10 L of 20% aqueous methanol (MeOH) at room temperature for 24 h and filtered, while 200 g of *C. zambesicus* was extracted with 2 L of the same solvent mixture. The crude extract from each species was separately concentrated *in vacuo* at 40 °C to about a third of the original volume. This afforded individual crude extract for each species.

#### 3.2.2. Solvent Portioning of the Crude Extracts

Concentrated crude extracts were in turn sequentially extracted with *n*-hexane, dichloromethane (DCM), ethyl acetate (EtOAc) and finally *n*-butanol. The solvent fractions were individually concentrated to dryness *in vacuo* to afford four solvent fractions each. The crude extracts and fractions of both species were separately subjected to various antioxidant and the AChE inhibitory bioassays (described later in this article) in which the ethyl acetate fractions from both species were selected for isolation of active principles.

### 3.3. Isolation and Identification of Compounds

#### 3.3.1. *C. zambesicus* (subgratissimus) Ethyl Acetate Fraction

Isolation and identification of compounds **1**–**3** from the ethyl acetate fraction of *C. zambesicus* were as previously described by Aderogba *et al.* [31]. The compounds are: quercetin-3-*O*-β-6’’(p-coumaroyl) glucopyranoside-3’-methyl ether, helichrysoside-3’-methyl ether (**1**), kaempferol-3-*O*-β-6’’(*p*-coumaroyl)glucopyranoside, tiliroside (**2**) and apigenin-6-*C*-glucoside, isovitexin (**3**).

#### 3.3.2. *C. gratissimus* Ethyl Acetate Fraction

The EtOAc fraction (2.0 g) was fractionated on a Sephadex LH-20 column using 93% DCM/MeOH as eluent. This was followed by an increasing gradient of methanol up to 40%. The fractions collected were analysed by TLC using DCM/MeOH (8.5:1.5) as a solvent system. Fraction A_2_ (800 mg) was subjected to column chromatograph on Sephadex LH-20 using EtOAc /MeOH (9:1) as eluent followed by an increasing gradient of methanol up to 20%. Analysis of the fractions collected on TLC plates using DCM/MeOH (8.5:1.5) yielded four fractions B_1_–B_4_. Fraction B_2_ had one major spot and was purified on Sephadex LH-20 column using 90% CHCl_3_/MeOH as eluent. This gave compound **2** (10 mg, tiliroside, Aderogba *et al*., [31]). Purification of fraction B_3_ on Sephadex LH-20 using CHCl_3_/MeOH (4:1) and analysis of the fractions by TLC using CHCl_3_/MeOH (7:3) afforded compound **3** (9 mg, isovitexin [31]. Purification of fraction B_1_ on preparative TLC plates using CHCl_3_/MeOH (7:3) yielded compound **4** (3 mg, kaempferaol, [32]).

#### 3.3.3. Compounds Combinations for the Test

Three of the isolated compounds (**1**–**3**) were mixed to make different combinations as follows: combination 1 (helichrysoside-3’-methyl ether + tiliroside + isovitexin, v/v/v), combination 2 (helichrysoside-3’-methyl ether + tiliroside, v/v), combination 3 (helichrysoside-3’-methyl ether + isovitexin, v/v) and combination 4 (tiliroside + isovitexin, v/v). Compound 4 was isolated in too small a quantity (3 mg) and was not tested in the assays. Bioassays were performed on each of these isolated compounds as well as their four combinations.

### 3.4. Bioassays

#### 3.4.1. DPPH Radical Scavenging Activity

The DPPH radical scavenging assay was performed according to a method described by Karioti *et al*. [33] with modifications. Fifteen microlitres of each test sample (0.065, 0.26, 0.52, 1.04, 6.25, 12.5, 25 and 50 mg/mL for crude extracts and 0.0625, 0.125, 0.25, 0.5, 1 mg/mL for compounds, in triplicate), was diluted in methanol (735 µL) and added to freshly prepared methanolic DPPH solution (750 µL, 50 µM) to give a final volume of 1.5 mL in the reaction mixture. The reaction preparations were prepared under dim light and incubated at room temperature for 30 min in the dark. Absorbance was read at 517 nm using a UV-vis spectrophotometer (Varian Cary 50, Melbourne, Australia), with methanol as the blank solution. A standard antioxidant, ascorbic acid (5, 10, 20, 40, 80 µM) was used as a positive control. A solution with the same chemicals without test sample or standard antioxidants served as the negative control. The assay was repeated three times. The free radical scavenging activity (RSA) as determined by the decolouration of the DPPH solution was calculated according to the formula:

$$\mathrm{RSA}\;\left(\%\right)=\left\{1\;-\;\left(\frac{{\mathrm{Abs}}_{517\;\mathrm{nm}}\;\mathrm{Sample}\;}{{\mathrm{Abs}}_{517\;\mathrm{nm}}\;\mathrm{Neg}\;\mathrm{Control}}\right)\right\}\;\times \;100$$

where Abs_517_ sample is the absorbance of the reaction mixture containing the test sample or positive control solution, and Abs_517_ Neg control is the absorbance of the negative control. The EC_50_ (effective concentration) values, representing the amount of extract required to decrease the absorbance of DPPH by 50% was calculated from the percentage radical scavenging activity.

#### 3.4.2. Ferric-Reducing Power Assay

The ferric reducing power of the test samples was determined based on the method by Lim *et al*. [34] with modifications. Thirty microlitres of each test sample (6.25 mg/mL for crude extracts and 1 mg/mL for isolated compounds) and the positive control (ascorbic acid dissolved in methanol) was added to a 96 well microtitre plate in triplicate and two-fold serially diluted down the wells of the plate. To each well, 40 µL potassium phosphate buffer (0.2 M, pH 7.2) and 40 µL potassium ferricyanide (1% in phosphate buffer, w/v) were added. The microtitre plate was covered with foil and incubated at 50 °C for 20 min. After the incubation period, 40 µL trichloroacetic acid (10% in phosphate buffer, w/v), 150 µL distilled water and 50 µL FeCl_3_ (0.1% in phosphate buffer, w/v) were added. The microtitre plate was re-covered with foil and incubated at room temperature for 30 min. Absorbance was measured at 630 nm using a microtitre plate reader (Opsys MR^TM^, Dynex Technologies Inc., Virginia, USA). The ferric-reducing power of the test samples and ascorbic acid were expressed graphically by plotting absorbance against concentration. The assay was repeated three times.

#### 3.4.3. β-Carotene-Linoleic Acid Model System (CLAMS)

The delay or inhibition of *β*-carotene and linoleic acid oxidation was measured according to the method described by Amarowicz *et al*. [35] with modifications. The antioxidant assay measures the ability of a test solution to prevent or minimize the coupled oxidation of *β*-carotene and linoleic acid in an emulsified aqueous system. In the reaction, the emulsion loses its orange colour due to the reaction with radicals, but this process can be inhibited by antioxidants.

*β*-Carotene (10 mg) was dissolved in 10 mL chloroform in a brown Schott bottle. The excess chloroform was evaporated under vacuum, leaving a thin film of *β*-carotene near to dryness. Linoleic acid (200 µL) and Tween 20 (2 mL) were immediately added to the thin film of *β*-carotene and mixed with aerated distilled water (497.8 mL), giving a final *β*-carotene concentration of 20 µg/mL. The mixture was further saturated with oxygen by vigorous agitation to form an orange coloured emulsion. The emulsion (4.8 mL) was dispensed into test tubes to which the crude extracts and compounds (200 µL of 6.25 mg/mL and 1 mg/mL respectively) were added, giving a final concentration of 250 µg/mL and 20 µg/mL for crude extracts and isolated compounds respectively, in the reaction mixtures. Absorbance for each reaction was immediately (*t* = 0) measured at 470 nm and incubated at 50 °C, with absorbance of each reaction mixture being measured every 30 min for 180 min. Tween 20 solution was used to blank the spectrophotometer. The negative control consisted of 50% methanol in place of the sample. The rate of *β*-carotene bleaching was calculated using the following formula:

$$\mathrm{Rate}\;\mathrm{of}\;\mathrm{bleaching}\;(R)=\left\{\ln\left(\frac{A_{t=0}}{A_{t=t}}\right)\right\}\;\times \;\frac{1}{t}$$

where *A_t=0_* is the absorbance of the emulsion at 0 min; and *A_t=t_* is the absorbance at time *t* (90 min; any point on the curve can be used for the calculation). The calculated average rates were used to determine the antioxidant activity (ANT) of the respective samples, and expressed as percentage of inhibition of the rate of *β*-carotene bleaching using the formula:

$$\%\;\mathrm{ANT}=\left(\frac{R_{\;\mathrm{control}\;}-{\;R}_{\;\mathrm{sample}\;}}{R_{\;\mathrm{control}\;}}\right)\times \;100$$

where R_control_ and R_sample_ represent the respective average *β*-carotene bleaching rates for the control and test samples, respectively. Antioxidant activity was further expressed as the oxidation rate ratio (ORR) based on the equation:

$$\mathrm{ORR}=\frac{R_{\;\mathrm{sample}\;}}{R_{\;\mathrm{control}\;}}$$

#### 3.4.4. Phospholipid Peroxidation Assay Coupled with the Thiobarbituric Acid-Malondialdehyde (TBA-MDA) Model System for Estimation of Oxidative Protection

The estimation of oxidative protection of lipids by the test samples in a model system was carried out using a coupled phospholipid peroxidation assay and the thiobarbituric acid-malondialdehyde (TBA-MDA) model system. In biological systems, lipid peroxidation generates a number of degradation products such as MDA which has been used as a maker of oxidative stress. This method follows, spectrophotometrically the production of MDA as a result of lipid peroxidation after a specific time period.

Rat brain tissue (2 g), previously stored at −70 °C was homogenised in 10 mL of chloroform:MeOH (2:1, v/v) using a homogeniser followed by centrifugation at 3,000 rpm for 5 min. The supernatant obtained was used as the phospholipid solution.

To test tubes (two sets, A and B in duplicate) containing 100 µL of test sample (10 mg/mL crude extract and 1 mg/mL isolated compounds to obtain a final concentration of 3.5 mg/mL and 350 µg/mL respectively), 50 µL of the phospholipid solution was added followed by 200 µL of methanol and 500 µL of FeSO_4_ (0.1 mM) (FeSO_4_ served as the primmer for peroxidation). The mixture was incubated for 30 min at 37 °C. The amount of MDA produced during the incubation period was measured using the TBA-MDA assay as described by Hodges *et al.* [27], as outlined below.

To the reaction mixture set A, taken from the 37 °C incubator, 650 µL of +TBA solution consisting of 20% trichloroacetic acid (TCA) and 0.65% TBA was added while –TBA solution consisting of only 20% TCA was added to set B. The tubes were mixed vigorously and heated at 95 °C in a heat block for 25 min. The tubes were allowed to cool and the contents were centrifuged at 3,000 rpm for 10 min. The absorbance of the supernatant was read at 440 nm, 532 nm and 600 nm. MDA equivalents were calculated in the manner:

$$A=\left\{\left(\mathrm{Abs}\;532_{+\mathrm{TBA}}-\mathrm{Abs}\;600_{+\mathrm{TBA}}\;)-(\mathrm{Abs}\;532_{-\mathrm{TBA}}-\mathrm{Abs}\;600_{-\mathrm{TBA}}\right)\right\}$$

$$B=\left(\mathrm{Abs}\;400_{+\mathrm{TBA}}-\mathrm{ABS}\;600_{+\mathrm{TBA}}\right)$$

$$\mathrm{MDA}\;\mathrm{equivalents}\;(\mathrm{nmol}/\mathrm{mL})=\left(\frac{A-B}{157000}\right)\;\times \;106$$

where Abs 532_+TBA_, Abs 600_+TBA_ and Abs 440_+TBA_ is the absorbance of the sample at a specified wavelength for test tube set A. Abs 532_−TBA_, Abs 600_−TBA_ and Abs 440_−TBA_ is the absorbance of the sample at a specified wavelength for test tube set B. The value 157 000 was used as the molar extinction coefficient for MDA. This assay increases the accuracy of determining TBA-MDA levels by correcting for compounds other than MDA which absorbs at 532 nm by subtracting the absorbance at 532 nm of a solution containing test sample incubated without TBA from an identical solution containing TBA. Lower amounts of MDA, signifies higher protective ability of extract against lipid peroxidation.

#### 3.4.5. Acetylcholinesterase (AChE) Enzyme Inhibitory Bioassay

Inhibition of AChE enzyme by the extracts and compounds was done as described by Ellman *et al.* [36] with some modifications as detailed by Moyo *et al*. [37]. The final concentration of the test samples in the first well containing the highest concentration was 1.0 mg/mL for crude extracts and 250 µg/mL for compounds. Galanthamine at 0.12, 0.23, 0.46, 0.92, 1.84, 3.68 and 7.37 µg/mL concentrations and water were used as positive and negative controls respectively. The microplate assay utilised an Opsys MR 96-well microplate reader. In a 96-well plate, 25 µL of 15 mM ATCI in water, 125 µL of 3 mM DTNB in buffer C (50 mMTris-HCl, pH 8, containing 0.1 M NaCl, 0.02 M MgCl_2_.6H_2_O), 50 µL of buffer B (50 mMTris-HCl, pH 8, containing 0.1% bovine serum albumin), 25 µL of sample dissolved in 50% aqueous methanol were added and the absorbance was measured at 405 nm every 45 s (five times). 25 µL of 0.2 U/mL of enzyme (AChE from electric eel Type VI-s, Sigma chemical Co.) were added and the absorbance measured again every 45 s (eight times). The rate of reaction was calculated. Any increase in absorbance due to the spontaneous hydrolysis of the substrate was corrected by subtracting the rate of reaction before adding the enzyme from the rate after adding the enzyme. Percentage of inhibition was calculated by comparing the rates for the samples to the blank (10% methanol in buffer A). Results of all the assays were presented as means ± standard errors. The IC_50_ values of test samples were calculated using GraphPad Prism (version 4.0) statistical software programme for Windows (GraphPad Software Inc., California, USA).

## 4. Conclusions

Oxidative damage has been implicated as the key factor in accelerated pathogenesis of a number of human diseases including cardiovascular, inflammatory, cancer and neurodegenerative diseases. Natural plant-derived chemotherapies, particularly phenolic compounds have demonstrated to be good antioxidants. *Croton gratissimus* ethyl acetate and butanol fractions, among the crude extracts, exhibited good antioxidant and acetylcholinesterase inhibitory activities in this study. Combinatorial chemotherapy treatment strategies have emerged to be one of the most useful in the treatment of diverse ailments. The results of combining the isolated compounds in this study also proved to have an increased efficacy when compared to the activities of the individual compounds assessed independently. The geographical location of plant species also seem to have a profound effect on the concentrations of the active constituents in the extracts as indicated by the differences in the efficacy of the two *Croton* species in this study despite their similarities in the phytochemistry.

## Figures and Tables

**Table 1:** Antioxidant activity as determined by the DPPH scavenging assay and *β*-carotene-linoleic acid model system of the isolated compounds, crude extracts and fractions from *C. gratissimus* and *C. zambesicus*. n = 3.

Sample	Antioxidant capacity		
	DPPH scavenging activity EC_50_ (µg/mL)	ANT (%)	ORR
Helichrysoside-3’-methyl ether (**1**)	183.35 ± 17.15	82.63 ± 0.85	**0.17 ± 0.01**
Tiliroside (**2**)	360.05 ± 4.95	93.84 ± 0.38	**0.06 ± 0.01**
Isovitexin (**3**)	211.55 ± 5.85	74.74 ± 0.01	0.25 ± 0.01
Helichrysoside-3’-methyl ether + tiliroside + isovitexin (1:1:1, v/v/v)	**65.23 ± 1.44**	84.99 ± 0.72	**0.15 ± 0.01**
Helichrysoside-3’-methyl ether + tiliroside (1:1, v/v)	95.33 ± 5.60	77.95 ± 0.10	0.22 ± 0.01
Helichrysoside-3’-methyl ether + isovitexin (1:1, v/v)	**68.70 ± 1.45**	87.20 ± 3.76	**0.13 ± 0.04**
Tiliroside + isovitexin (1:1, v/v)	113.40 ± 2.39	72.43 ± 2.62	0.28 ± 0.03
*Croton gratissimus* crude extract (20% MeOH)	**58.47** ± **5.06**	94.18 ± 0.85	**0.06 ± 0.01**
*Croton gratissimus* hexane	107.92 ± 13.29	80.61 ± 1.47	**0.19 ± 0.01**
*Croton gratissimus* DCM	749.00 ± 15.00	67.12 ± 0.99	0.33 ± 0.01
*Croton gratissimus* ethyl acetate	**36.86** ± **0.61**	96.66 ± 2.067	**0.03 ± 0.02**
*Croton gratissimus* butanol	**11.15** ± **0.59**	95.72 ± 0.10	**0.04 ± 0.00**
*Croton zambesicus* crude extract (20% MeOH)	1018.15 ± 55.85	83.18 ± 0.88	**0.17 ± 0.01**
*Croton zambesicus* hexane	2894.00 ± 26.00	78.49 ± 1.89	0.22 ± 0.02
*Croton zambesicus* DCM	1673.00 ± 13.70	84.90 ± 0.49	**0.15 ± 0.01**
*Croton zambesicus* ethyl acetate	970.05 ± 16.45	92.19 ± 0.08	**0.08 ± 0.00**
*Croton zambesicus* butanol	740.2 ± 0.90	87.54 ± 2.019	**0.12 ± 0.02**
Ascorbic acid	70.12 ± 0.01	81.45 ± 1.72	0.19 ± 0.02

Compounds/extracts with EC_50_ values (<70.12 µg/mL) in bold are considered potent DPPH radical scavengers. The lower the EC_50_, the more rapidly the colour of DPPH radical was bleached and hence the more potent the antioxidant. ANT (%): Antioxidant activity calculated on the basis of the rate of *β*-carotene bleaching at *t* = 90 min. ORR: Oxidation Rate Ratio at *t* = 90. The lower the ORR value, the more protective the compound/extract against *β*-carotene bleaching. Compounds/extracts with ORR values (≤0.19) in bold are considered potent antioxidants. Values represent mean ± standard error (n = 3).

**Table 2:** AChE inhibitory activity (IC_50_ µg/mL) of the isolated compounds, compound combinations, crude extracts and fractions from *C. gratissimus* and *C. zambesicus*. Values represent mean ± standard error (n = 3).

Sample	AChE inhibitory activity	
	% Inhibition	IC_50_ (µg/mL)
Helichrysoside-3’-methyl ether	11.4 ± 0.1 ^a^	787.2 ± 2.6
Tiliroside	31.8 ± 2.1 ^a^	391.3 ± 0.2
Isovitexin	52.1 ± 3.2 ^a^	189.5 ± 2.7
Helichrysoside-3’-methyl ether + tiliroside + isovitexin (1:1:1, v/v/v)	67.1 ± 5.1 ^b^	267.2 ± 4.0
Helichrysoside-3’-methyl ether + tiliroside (1:1, v/v)	68.8 ± 3.0 ^b^	200.2 ± 2.2
Helichrysoside-3’-methyl ether + isovitexin (1:1, v/v)	67.3 ± 4.4 ^b^	187.9 ± 3.6
Tiliroside + isovitexin (1:1, v/v)	74.4 ± 0.1 ^b^	141.7 ± 3.4
*Croton gratissimus* crude extract (20% MeOH)	98.7 ± 5.1 ^c^	208.3 ± 1.0
*Croton gratissimus* hexane	92.1 ± 1.0	307.1 ± 12.1
*Croton gratissimus* DCM	66.1 ± 2.1	537.7 ± 3.9
*Croton gratissimus* ethyl acetate	70.9 ± 3.4	**66.9 ± 2.4**
*Croton gratissimus* butanol	71.8 ± 2.2	**64.5 ± 5.2**
*Croton zambesicus* crude extract (20% MeOH)	51.4 ± 7.2	282.1 ± 4.1
*Croton zambesicus* hexane	49.8 ± 1.2	387.4 ± 3.4
*Croton zambesicus* DCM	31.9 ± 3.7	469.7 ± 1.1
*Croton zambesicus* ethyl acetate	54.2 ± 2.2	256.8 ± 4.2
*Croton zambesicus* butanol	61.4 ± 4.5	449.1 ± 4.9
Galanthamine	87.9 ± 2.4	± 0.6 (µM)

Test samples with IC_50_ values in bold are considered potent inhibitors of AChE. ^a^, % inhibition at compound concentration of 250 µg/mL; ^b^, % inhibition at individual concentration of 250 µg/mL; ^c^, % inhibition of crude extracts and fractions at concentration of 650 µg/mL.
